# Correction: Wang et al. Casein Kinase 1α as a Novel Factor Affects Thyrotropin Synthesis via PKC/ERK/CREB Signaling. *Int. J. Mol. Sci.* 2023, *24*, 7034

**DOI:** 10.3390/ijms27115015

**Published:** 2026-06-02

**Authors:** Bingjie Wang, Jinglin Zhang, Di Zhang, Chenyang Lu, Hui Liu, Qiao Gao, Tongjuan Niu, Mengqing Yin, Sheng Cui

**Affiliations:** 1 College of Veterinary Medicine, Yangzhou University, Yangzhou 225009, China; 2 Key Laboratory of Animal Medicine of Sichuan Education Department, Southwest Minzu University, Chengdu 610041, China; 3 Institute of Reproduction and Metabolism, Yangzhou University, Yangzhou 225009, China; 4 Joint International Research Laboratory of Agriculture and Agri-Product Safety, The Ministry of Education of China, Yangzhou University, Yangzhou 225009, China; 5 Jiangsu Co-Innovation Center for Prevention and Control of Important Animal Infectious Diseases and Zoonoses, Yangzhou 225009, China

**Missing Funding**

In the original publication [1], funding support from the Sichuan Science and Technology Program (2025ZNSFSC1075) and the Southwest Minzu University Research Startup Funds (Grant no. RQD2025017) to Wang et al. were not included. The corrected Funding section part appears below.

**Funding**: This research was funded by the Natural Science Foundation of China (32130098 and 32102618), Priority Academic Program Development of Jiangsu Higher Education Institutions (PAPD), Sichuan Science and Technology Program (2025ZNSFSC1075) and Southwest Minzu University Research Startup Funds (RQD2025017).

**Additional Affiliation**

There was an error regarding the affiliation of Bingjie Wang. In addition to affiliation 1, the updated affiliation should include: 2. Key Laboratory of Animal Medicine of Sichuan Education Department, Southwest Minzu University, Chengdu 610041, China. 

**Figure Legend**

There was a mistake in Figure 1b as published. The band of CK1α is absent in the Western Blot of Figure 1b. The corrected Figure 1 appears below. 

The authors state that the scientific conclusions are unaffected. These corrections were approved by the Academic Editor. The original publication has also been updated.

## Figures and Tables

**Figure 1:**
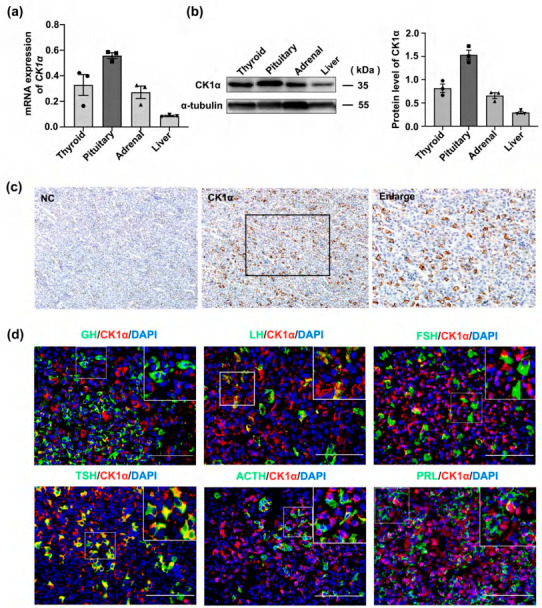
CK1α is expressed in murine pituitary thyrotropes. (**a**) The expression of CK1α in different mouse tissues was detected using real-time PCR (RT-PCR) and normalized to Gapdh expression. Data are presented as means ± standard error of the mean of three stand-alone experiments. (**b**) CK1α protein expression in different mouse tissues was analyzed using Western blotting and normalized to α-tubulin expression; *n* = 3. (**c**) The expression of CK1α in pituitary tissue was detected using immunohistochemistry. Scale bar = 100 μm. (**d**) Detection of CK1α expression in different pituitary cell types using immunofluorescence chemistry. Scale bar = 100 μm. Each experiment was independently repeated three times.
