# Supplementary material for: Casein Kinase 1α as a Novel Factor Affects Thyrotropin Synthesis via PKC/ERK/CREB Signaling
Source: Int J Mol Sci. 2023 Apr 11;24(8):7034. doi: 10.3390/ijms24087034 (PMC10138882; doi:10.3390/ijms24087034)
Supplement: Supplementary file 1 [file ijms-24-07034-s001.zip › ijms-2279573-supplementary.pdf]

SUPPLEMENTARY MATERIALS

Table S1. Weight of mice (mean ± SD, n = 5)

Group	Weight (g)							
	D0	D2	D4	D6	D8	D10	D12	D14
Control-1	21.28±2.87	21.40±2.86	21.14±2.81	21.17±2.73	21.41±2.63	21.52±2.71	22.84±2.85	23.06±3.01
D4476 (1 mg/mL)	22.31±1.30	22.42±1.36	21.70±1.69	21.91±1.26	22.34±1.06	21.75±0.78	22.28±0.65	23.37±1.06
Control-2	31.88±0.76	31.65±0.67	30.65±1.36	30.32±1.43	30.16±1.57	30.12±1.94	30.86±1.39	31.32±1.34
Pyrvinium (5 mg/mL)	32.43±0.62	32.23±0.68	31.36±0.82	31.37±1.03	30.7±1.22	30.12±0.88	30.58±1.03	30.88±1.09

Table S2. Visceral ratio (mean ± SD, n = 5)

Group	Heart (g)	Liver(g)	Kidney(g)	Testis(g)
**Control-1**	5.3118±0.63605	37.7322±1.76314	15.3818±2.05882	8.4952±1.02917
**D4476 (1 mg/mL)**	4.9614±0.37106	37.4405±2.56482	14.3418±0.45137	8.7220±0.54971
**Control-2**	4.5606±0.28835	38.2290±2.65378	11.9062±2.19537	9.9866±1.63244
**Pyrvinium (5 mg/mL)**	4.6494±0.98788	39.4565±2.20680	12.2400±0.71699	9.8987±0.43588

Table S3. Organ coefficient (mean ± SD, n = 5)

Group	Cardiac index (mg/g)	Liver index (mg/g)	Kidney index (mg/g)	Testicular index (mg/g)
**Control-1**	0.2360 ± 0.04803	1.6575 ± 0.15304	0.6787 ± 0.09503	0.3797 ± 0.09654
**D4476 (1 mg/mL)**	0.1819 ± 0.01572	1.4869 ± 0.11394	0.5249 ± 0.02599	0.3607 ± 0.02835
**Control-2**	0.1459 ± 0.01154	1.2238 ± 0.11223	0.3795 ± 0.06706	0.3205 ± 0.06170
**Pyrvinium (5 mg/mL)**	0.1548 ± 0.0.0363	1.3271 ± 0.08385	0.4105 ± 0.03634	0.3173 ± 0.01310

Table S4. Primer sequences for RT-PCR.

Primer names	Sequence
Gapdh forward	5′-AGCAATGCCTCCTGCACCACCA-3′
Gapdh reverse	3′-TGAGTCCCTCCACGATGCCGAA-5′
Tshb forward	5′-GTGCTGGGTATTGTATGACACG-3′
Tshb reverse	3′-CTGGTATTTCCACCGTTCTGTAG-5′
Csnk1a1 forward	5′-GACCCAGCCTTGAAGACCTC-3′
Csnk1a1 reverse	3′-CAGTGACGCCCAATACCCAT-5′
Gh forward	5′-*AGAACCGACATGGAATTGCTT*-3′
Gh reverse	3′-*ATGTTGGCGTCAAACTTGTCA*-5′
Pomc forward	5′-GCAACGGAGATGAACAGCC-3′
Pomc reverse	3′-CTTGTCCTTGGGCGGGTT-5′
Prl forward	5′-GGAGAAGTGTGTTCCCAGC-3′
Prl reverse	3′-CAGCGAATGGTGTTGCGC-5′
Fsh forward	5′-CTGAATGTCACTGTGGCAAGT-3′
Fsh reverse	3′-GCAATGTCCATCGTCGTTTAT-5′
Lh forward	5′-CTGCCCAGTCTGCATCACC-3′
Lh reverse	3′-AGGCACAGGAGGCAAAGC-5′

Table S5. Antibodies used in the study

Antibody	Dilution	Description	Company	CAT#
CK1α	1:250 IHC, 1:2000 WB	Rabbit-polyclonal	Abcam	ab64939
α-Tubulin	1:10000 WB	Rabbit-polyclonal	Beyotime	AF0001
CK1α	1:250 IF	Chicken-polyclonal	Novus Biologicals	NBP2-50030
TSH-b	1:200 IF	Rabbit-polyclonal	NIDDK	
GH	1:100 IF	Rabbit-monoclonal	Abcam	ab155276
PRL	1:150 IF	Rabbit-polyclonal	NHPP	
FSH-b	1:100 IF	Rabbit-polyclonal	NHPP	
LH-b	1:100 IF	Rabbit-polyclonal	NIDDK	
ACTH	1:200 IF	Rabbit-polyclonal	NHPP	
p-CK1α	1:2000 WB	Rabbit-polyclonal	Bioss ANTIBODIES	bs-12428R
Active-β-catenin	1:2000 WB	Rabbit-polyclonal	Cell Signaling Technology	8814
β-Catenin	1:2000 WB	Rabbit-polyclonal	Cell Signaling Technology	8480
p-PKC	1:1000 WB	Rabbit-polyclonal	Millipore	AB5467
PKC	1:1000 WB	Rabbit-polyclonal	Wanleibio	WL02234
p-ERK1/2	1:300 WB	Rabbit-polyclonal	Wanleibio	WLP1512
ERK1/2	1:500 WB	Rabbit-polyclonal	Wanleibio	WL01864
p-CREB	1:1000 WB	Rabbit-polyclonal	Abmart	T55043
CREB	1:1000 WB	Rabbit-polyclonal	Abmart	T55426
Cy3 donkey Anti-chicken	1:200 IF	Donkey-polyclonal	Jackson Immuno Research	703-165-155
Cy2 donkey Anti-rabbit	1:200 IF	Donkey-polyclonal	Jackson Immuno Research	711-225-152
Goat-anti-rabbit	1:200 IHC, 1:5000 WB	Goat-polyclonal	Jackson Immuno Research	111-005-003
Goat-anti-mouse	1:5000 WB	Goat-polyclonal	Jackson Immuno Research	111-005-003

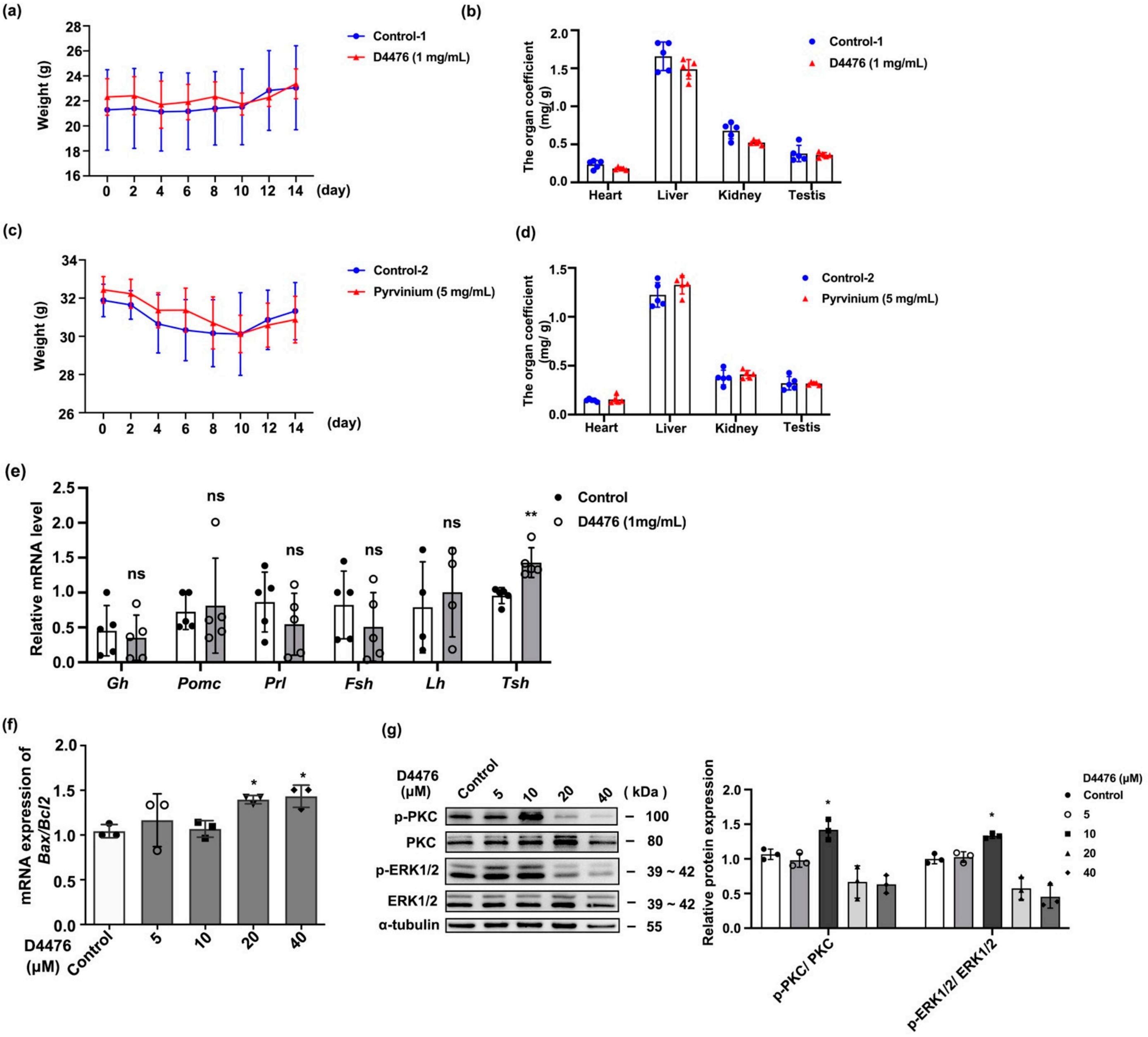

Figure S1. In vivo administration of D4476 and pyrvinium did not affect the key organ ratio and organ index. (**a, b**) D4476 (1 mg/mL) and pyrvinium (5 mg/mL) were administered for two weeks, and the weight change of mice was detected once every two days during the administration process. (**c, d**) After D4476 and PP administration for two weeks, key organs other than the pituitary gland, namely, the heart, liver, kidneys, and testes, were removed and the organ indices were measured. (**e**) After D4476 administration for two weeks, mRNA level of key pituitary hormone, namely, the *Gh* mRNA, *Pomc* mRNA, *Prl* mRNA, *Fsh* mRNA, *Lh* mRNA and the *Tsh* mRNA were measured by RT-PCR; $n = 5$. $^{**}P < 0.01$. (**f, g**) After *in vitro* pituitary primary cells were treated with different concentrations of D4476 (5, 10, 20, and 40 μM), quantitative levels of Bax/BCl2 were determined to assess the damage to cells at different concentrations and to screen for appropriate treatment concentrations; the phosphorylation level of key proteins in PKC/ERK/CREB signal pathway was detected by western blot. Relative p-PKC, p-ERK1/2, and p-CREB protein levels were normalized to those of total PKC, ERK1/2, and CREB; $n = 3$. $^{*}P < 0.05$.

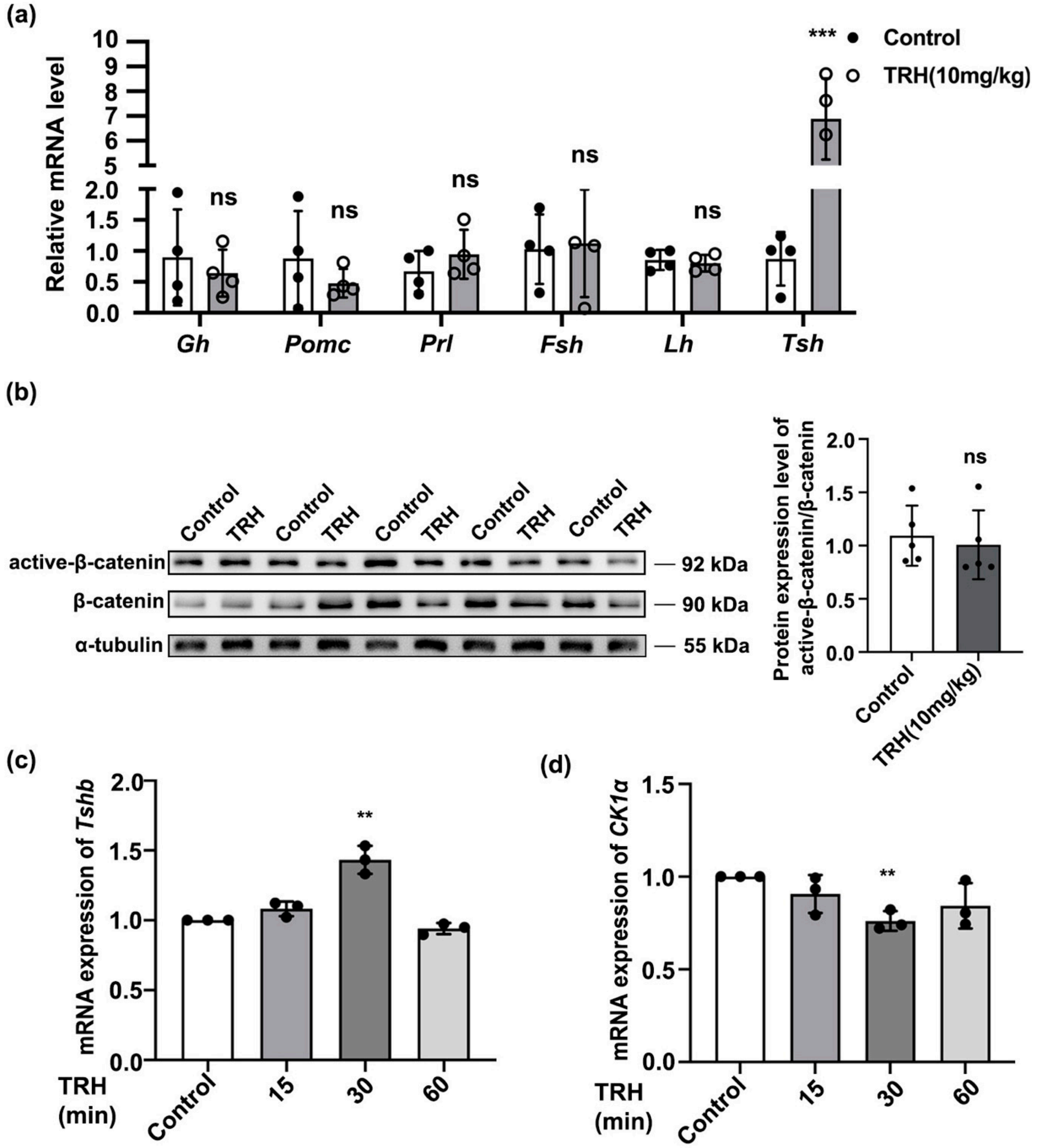

Figure S2. CK1α regulates the TRH-PKC/ ERK/ CREB signaling pathway and affects TSH synthesis. (**a**) After TRH administration for 30 min, mRNA level of key pituitary hormone, namely, the *Gh* mRNA, *Pomc* mRNA, *Prl* mRNA, *Fsh* mRNA, *Lh* mRNA and the *Tsh* mRNA were measured by RT-PCR; $n = 4$. ***$P < 0.001$. (**b**) The expression of Wnt/ b-catenin-related protein was detected after injection of 10 mg/kg TRH *in vivo*. Levels of active- β-catenin and β-catenin were analyzed by western blotting. The active- β-catenin/ β-catenin protein expression level was determined using densitometry. n = 3. (**c, d**) To screen out the appropriate TRH action time, we detected the levels of *Tshb* mRNA and *CK1a* mRNA by RT-PCR by treating pituitary primary cells with TRH for different times (15, 30, and 60 min). **$P < 0.01$.

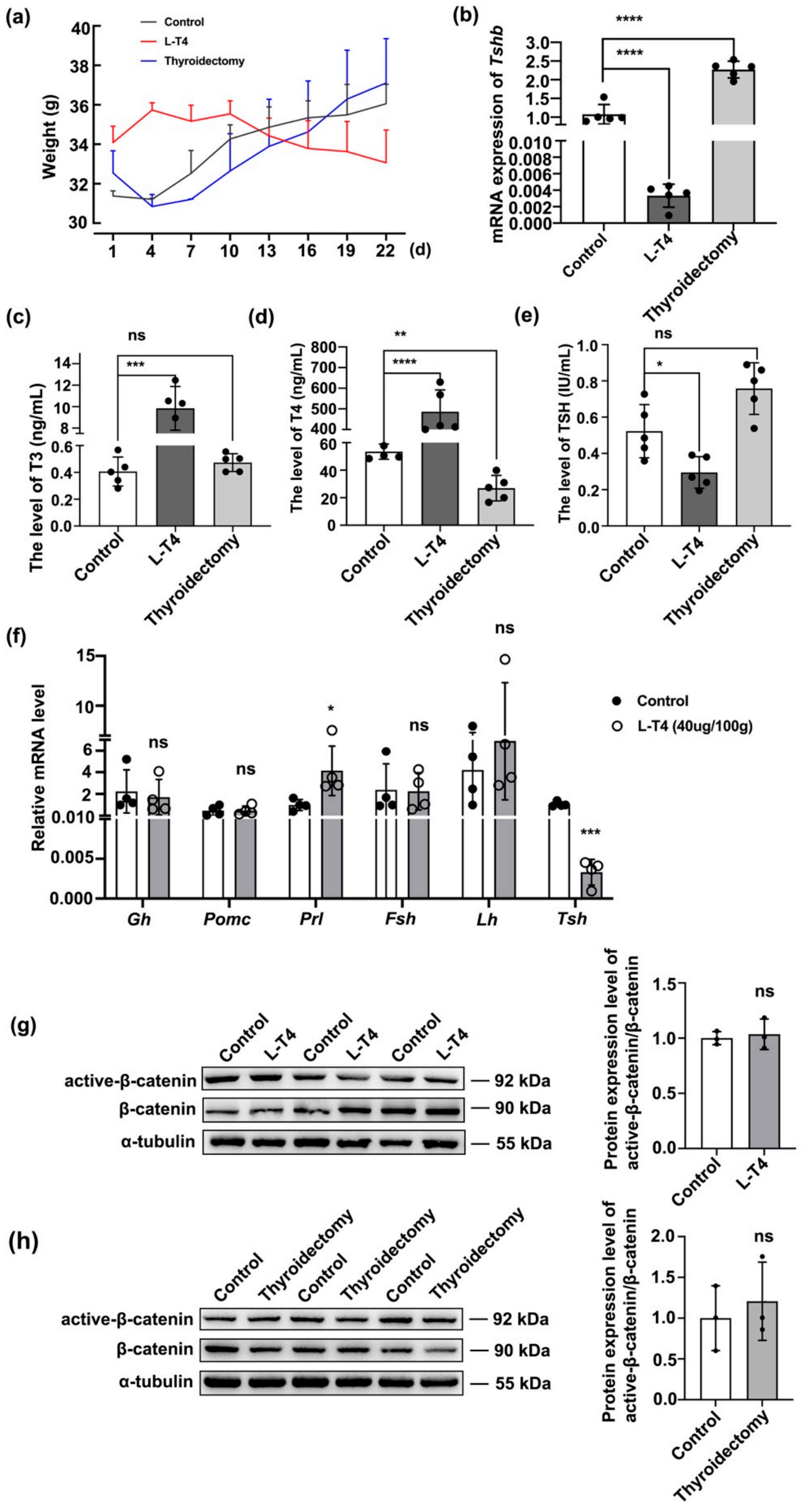

Figure S3. L-T4 enhances the activity of CK1*α*. **(a–e**) Negative feedback regulation of the HPT axis was simulated *in vivo*, and the synthesis and secretion of *Tshb* and TSH/T3/T4 were measured using RT-PCR and radioimmunoassay. **P* < 0.05, ***P* < 0.01, ****P* < 0.001, *****P* < 0.0001. (**f**) After L-T4 administration for 30 min, mRNA level of key pituitary hormone, namely, the *Gh* mRNA, *Pomc* mRNA, *Prl* mRNA, *Fsh* mRNA, *Lh* mRNA and the *Tsh* mRNA were measured by RT-PCR; *n* = 4. **P* < 0.05. **(g,h**) The effects of Wnt/b-catenin-related proteins were detected after *in vivo* injection of L-T4 and thyroidectomy; active- β-catenin and β-catenin expression levels were analyzed by western blotting. The active- β-catenin/ β-catenin protein expression level was determined using densitometry. n = 3.

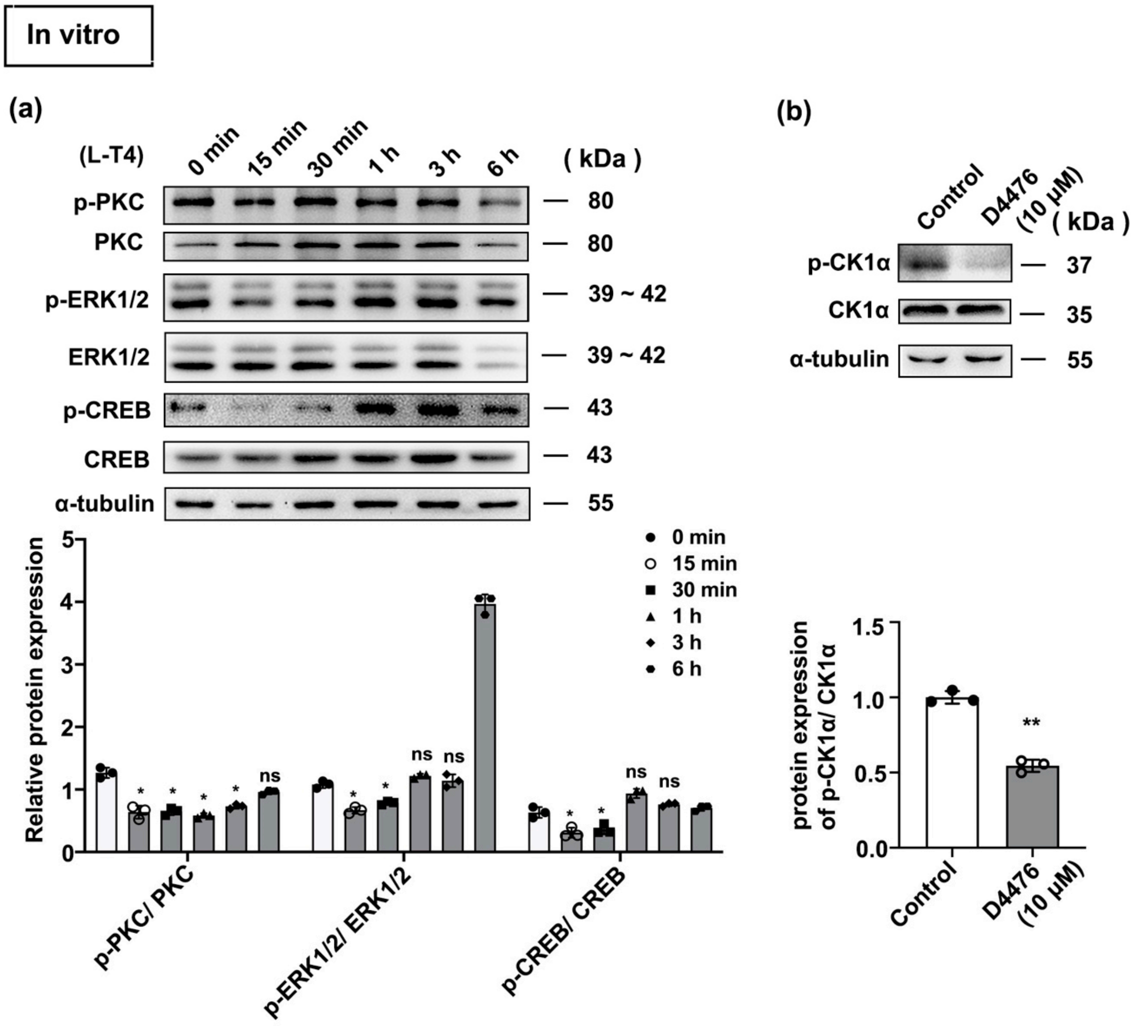

Figure S4. L-T4 inhibits TSH synthesis by promoting post-translational modification of CK1*α* to inhibit activation of the PKC/ERK/CREB pathway. (**a**) Primary pituitary cells were stimulated with 10 μM L-T4 for 0 min, 15 min, 30 min, 1 h, 3 h, or 6 h. Levels of p-PKC, and then p-ERK1/2, p-CREB, PKC, ERK1/2, and CREB levels were analyzed by western blotting. Relative p-PKC, p-ERK1/2, and p-CREB protein expression levels were determined using densitometry. n = 3. (**b**) The inhibition efficiency of CK1*α* was determined using western blotting after D4476 (10 μM) was added to primary cultured mouse adenohypophysis cells. *$P < 0.05$, **$P < 0.01$.

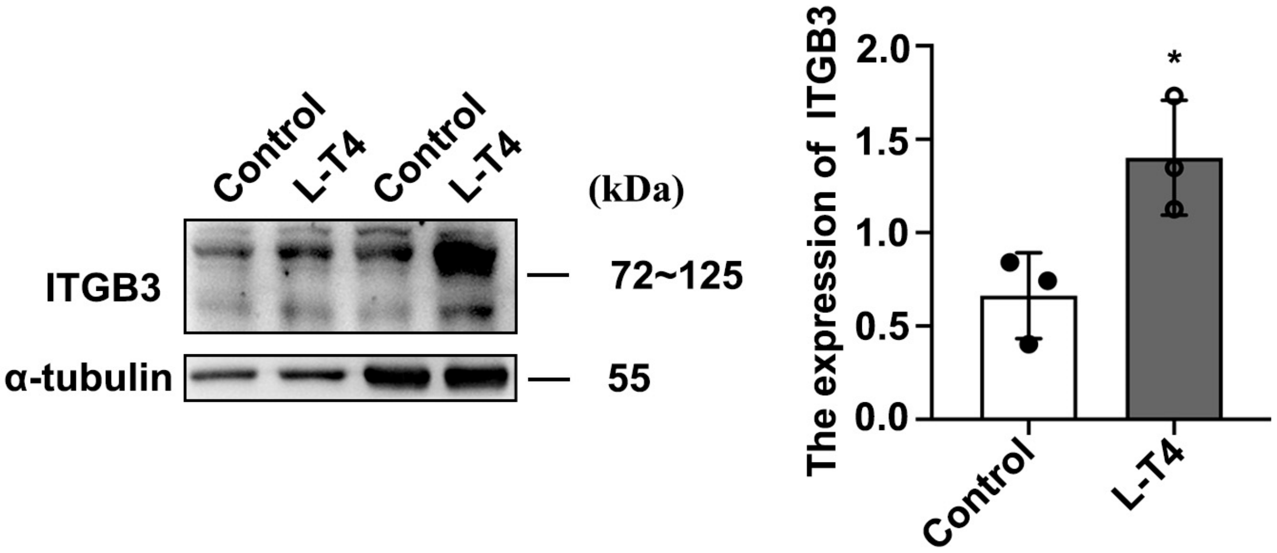

Figure S5. LT4 induces pituitary αVβ3 integrin receptor activation. Expression of ITGB3 was measured using western blotting with α-tubulin as an internal reference. *$P < 0.05$.
